# Chrysanthemum (*Chrysanthemum morifolium*) *CmHRE2-like* negatively regulates the resistance of chrysanthemum to the aphid (*Macrosiphoniella sanborni*)

**DOI:** 10.1186/s12870-024-04758-6

**Published:** 2024-01-29

**Authors:** You Wang, Wanwan Zhang, Chaojun Hong, Lisheng Zhai, Xinhui Wang, Lijie Zhou, Aiping Song, Jiafu Jiang, Likai Wang, Fadi Chen, Sumei Chen

**Affiliations:** 1 National Key Laboratory of Crop Genetics & Germplasm Enhancement and Utilization, College of Horticulture, Nanjing Agricultural University, Nanjing, P. R. China; 2 Key Laboratory of Flower Biology and Germplasm Innovation, Ministry of Agriculture and Rural Affairs, Beijing, P. R. China; 3 College of Horticulture, Nanjing Agricultural University, Nanjing, 210095 China

**Keywords:** Biotic, Flavonoid biosynthesis, Transcription, Aphid

## Abstract

**Background:**

The growth and ornamental value of chrysanthemums are frequently hindered by aphid attacks. The ethylene-responsive factor (ERF) gene family is pivotal in responding to biotic stress, including insect stress. However, to date, little is known regarding the involvement of ERF transcription factors (TFs) in the response of chrysanthemum to aphids.

**Results:**

In the present study, *CmHRE2-like* from chrysanthemum (*Chrysanthemum morifolium*), a transcription activator that localizes mainly to the nucleus, was cloned. Expression is induced by aphid infestation. Overexpression of *CmHRE2-like* in chrysanthemum mediated its susceptibility to aphids, whereas *CmHRE2-like*-SRDX dominant repressor transgenic plants enhanced the resistance of chrysanthemum to aphids, suggesting that *CmHRE2-like* contributes to the susceptibility of chrysanthemum to aphids. The flavonoids in *CmHRE2-like*-overexpression plants were decreased by 29% and 28% in two different lines, whereas they were increased by 42% and 29% in *CmHRE2-like*-SRDX dominant repressor transgenic plants. The expression of Chrysanthemum-chalcone-synthase gene(*CmCHS)*, chalcone isomerase gene (*CmCHI)*, and flavonoid 3′-hydroxylase gene(*CmF3’H*) was downregulated in *CmHRE2-like* overexpression plants and upregulated in *CmHRE2-like*-SRDX dominant repressor transgenic plants, suggesting that *CmHRE2-like* regulates the resistance of chrysanthemum to aphids partially through the regulation of flavonoid biosynthesis.

**Conclusion:**

*CmHRE2-like* was a key gene regulating the vulnerability of chrysanthemum to aphids. This study offers fresh perspectives on the molecular mechanisms of chrysanthemum-aphid interactions and may bear practical significance for developing new strategies to manage aphid infestation in chrysanthemums.

**Supplementary Information:**

The online version contains supplementary material available at 10.1186/s12870-024-04758-6.

## Background

Aphids damage plants by penetrating plant tissues and consuming phloem sap, thus depriving them of photosynthesis [1]. In order to prevent aphid infestations, plants employ a variety of direct and indirect defense strategies. For direct defense responses, plants respond to aphid infection by producing a range of different chemical elements, including extrafloral nectar, total flavonoids, latex, total phenolics, and tannins, which directly affect aphid growth and development [2]. Flavonoids are pivotal secondary metabolites in plants that play a significant role in enhancing their resistance to aphid infestation. For example, it has been found that soybean aphids behave differently depending on the flavonoid applied: apigenin, daidzein, and kaempferol reduce ingestion of plant sap by aphids [3]. Among endogenous flavonoids, quercetin and isorhamnetin have excellent inhibitory effects on aphid reproduction in cowpeas in vitro [4]. Cut-shoot bioassays were employed in apples to examine the efficacy of three flavonoids in combating woolly apple aphids [5]. However, the available literature on the influence of flavonoids on chrysanthemum resistance to aphids is limited.

The APETALA2/ethylene responsive factor (AP2/ERF) belongs to a large multigene family of transcription factors (TFs), it mediates several physiological, developmental, and stress-related responses [6]. This gene family encodes proteins with AP2/ERF DNA-binding domains, typically spanning 57–66 amino acids, which are known for their ability to bind to a range of cis-acting elements in the promoters of ethylene-responsive genes [7, 8] Several *ERF* family genes are recognized for their roles in modulating diverse responses to biotic stress. For example, five *ERF-B3* genes are involved in the response to the yellow leaf curly virus transmitted by the whitefly (*Bemisia tabaci*) [9]. Soybean-derived *GmERF3* belongs to the AP2/ERF TF family, its ectopic expression in transgenic tobacco plants resulted in heightened resistance to pathogens *Ralstonia solanacearum*, *Alternaria alternata*, and the tobacco mosaic virus (TMV) [10]. Overexpression of *GmERF5* enhances resistance to *Phytophthora* root and stem rot in soybeans [11]. Furthermore, *AtERF014* functions as a dual regulator, with distinct effects on the immunity against *Pseudomonas syringaepv. tomato* and *Botrytis cinerea* by affecting pectin content in *Arabidopsis* [12]. Similarly, the cabbage gene *BrERF11b* has been reported to be an ERF TF that enhances the resistance of plants against both the chewing insect *Spodoptera litura* and sap-sucking insect *Myzus persicae* [13]. In addition, the ERF TF *Pti5* exhibits increased expression when confronted with a potato aphid infestation., and silencing of the *PTI5* gene has been shown to reduce aphid resistance in tomatoes [14]. Nevertheless, as of our present understanding, there is a dearth of available data regarding the participation of ERF TFs in the response of chrysanthemum to aphid infection.

Chrysanthemum (*C. morifolium*) is prone to frequent attacks by aphids (*Macrosiphoniella sanborni*), resulting in significant yield losses and diminished ornamental value [15]. Aphids can deprive chrysanthemum of nutrients and transmit viruses [16]. Our earlier investigations have revealed that chrysanthemums have developed several strategies to cope with aphid infestation. *CmWRKY53* plays a role in mediating chrysanthemum’s susceptibility to aphids, potentially by regulating secondary metabolites [15]. *CmMYB15* enhances aphid resistance in chrysanthemum through the regulation of lignin biosynthesis genes [17] Overexpression of *CmMYB19* constrained aphid multiplication within the host plant by enhancing lignin accumulation [18]. *CmMYB15-like* expression is triggered by aphid infection leading to cell wall thickening and lignin deposition. As a result, aphid resistance is enhanced in a *Cm4CL2*-dependent manner [19]. Within this investigation, we isolated and characterized the *CmHRE2-like* gene in chrysanthemum and explored its function through the generation of *CmHRE2-like* transgenic plants. Our results show that *CmHRE2-like* modulates the vulnerability of chrysanthemum to aphids through the regulation of flavonoid biosynthesis. The current investigation offers fresh perspectives on the direct defense responses of chrysanthemums to aphids.

## Results

### Sequence analysis of *CmHRE2-like*

**Fig. 1:**
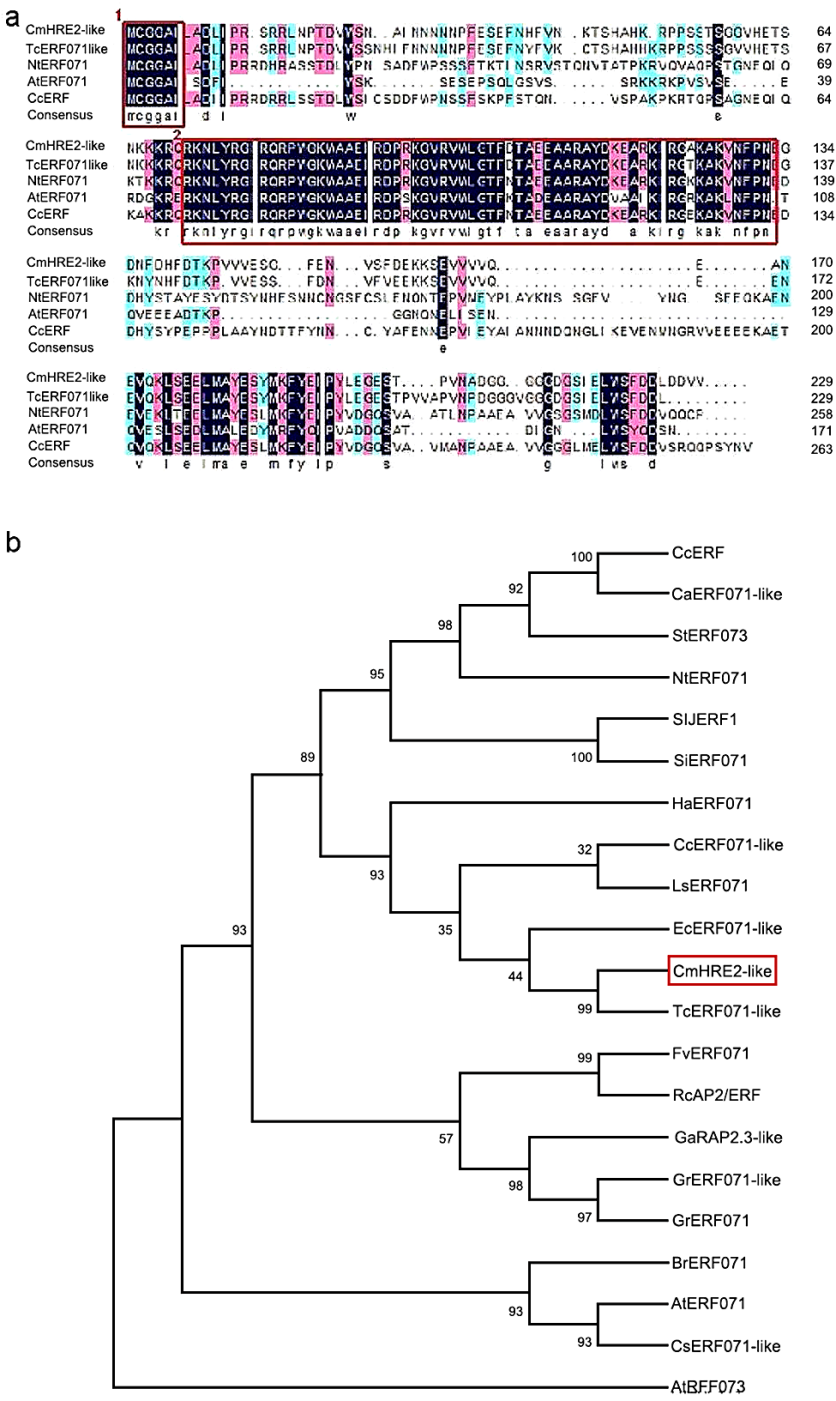
Amino acid sequence of CmHRE2-like and phylogenetic tree of ERF071s. **(a)** Comparative analysis of amino acids in CmHRE2-like and ERF071 homologs from various species. The regions highlighted in the red boxes represent the AP2/ERF domain (1) and the MCGGAII/L domain (2). **(b)** Phylogenetic tree of ERF071s. The regions highlighted in the red boxes represent *CmHRE2-like.* The sequence details are as follows: CcERF (AGU44838), CaERF071-like (NP_001311812.1), StERF073 (XP-006359865), NtERF071 (XP-009608570), SIJERF1 (NP-001234513), SiERF071 (XP-011079624), HaERF071 (XP_022035166.1), CcERF071-like (XP_024966446.1), LsERFO71 (XP_052622777.1), EcERF071-like (XP_043621228.1), TcERF071-like (GEU66948.1), FvERF071 (isoform X3 XP-004290818), RcAP2/ERF2 (protein AKC88471), GaRAP2-3-like (KHG20924), GrERF071-like (isoform X1 XP-012474966), GrERF071 (XP_012474966.1), BrERF071 (XP-009118731), AtERF071 (NP-182,274), CsERF071-like (XP-010518456), AtERF073 (NP-001117587)

*CmHRE2-like* containing a 678 bp open reading frame was isolated from chrysanthemum ‘Jinba’. *CmHRE2-like* encodes a polypeptide of 225 aa residues with a preserved DNA-binding domain recognized as AP2/ERF, and an N-terminal motif of undetermined function, designated as MCGGAII/L is present in *CmHRE2-like* (Fig. 1a). The function of the conserved N-terminal sequence, which represents a distinctive motif in several ERF family members, remains unclear [20], and phylogenetic analysis revealed that the sequence of *CmHRE2-like* bears the closest resemblance to *Tanacetum cinerariifolium TcERF071-like* (Fig. 1b). While preparing this manuscript, it was also noted that *AtHRE2* had been reported as ERF071.

### CmHRE2-like localized in nucleus and exhibited transcription activity

**Fig. 2:**
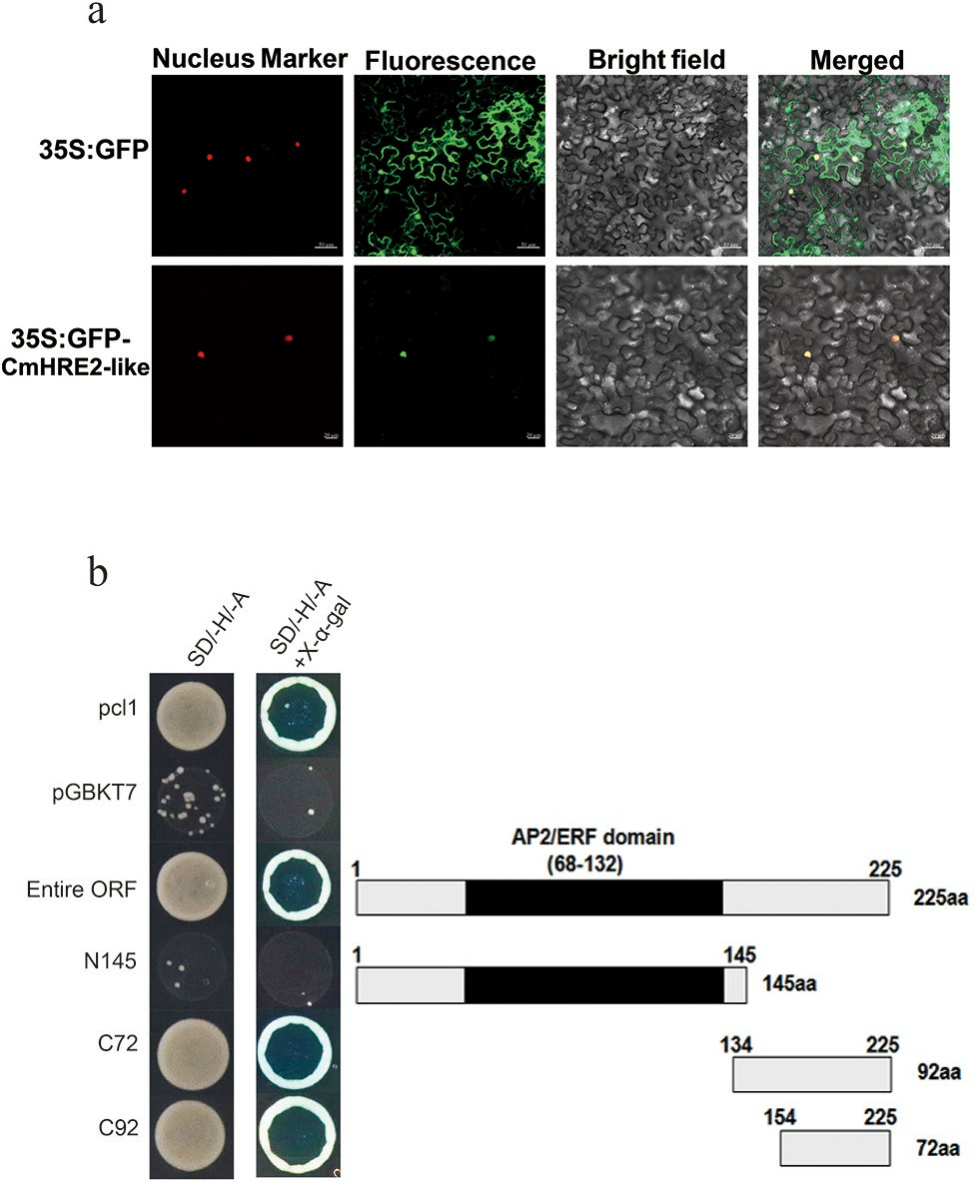
Analysis of the fundamental characteristics of *CmHRE2-like*. **(a)** Subcellular localization of *CmHRE2-like* in tobacco leaf cells. **(b)** Transactivation assay of *CmHRE2-like*

To characterize the sub-cellular localization of *CmHRE2-like*. The leaves of *Nicotiana benthamiana* were infiltrated with *Agrobacterium tumefaciens*, which was transformed with either pMDC43-GFP-*CmHRE2-like* or pMDC43-GFP empty vector. In tobacco cells infiltrated with the pMDC43-GFP-*CmHRE2-like* fusion protein, GFP fluorescence was observed solely within the nucleus, whereas tobacco cells infiltrated with pMDC43-GFP displayed an even distribution of GFP fluorescence (Fig. 2a), indicating that CmHRE2-like localized to the nucleus.

To assess the transcriptional activity of CmHRE2-like, the yeast strain Y2H was transformed with the pDEST-GBKT7-*CmHRE2-like* plasmid. The Y2H Gold yeast harboring either pDEST-GBKT7-*CmHRE2-like* construct and the pCL1 grew normally in SD/-His-Ade medium, while the yeast harboring negative control pDEST-GBKT7 showed no growth (Fig. 2b). Such observations indicate that the entire CmHRE2-like protein exhibited transcriptional activity. By cloning truncated segments from the C- and N-termini of CmHRE2-like into the pDEST-GBKT7 vector, the specific transactivation region of the protein was determined. In the double-deficient medium, yeast strains with the pDEST-GBKT7-*CmHRE2-like* (1–145 aa) didn’t proliferate, in contrast, fragments of 154–225 aa and the remaining sections exhibited normal growth (Fig. 2b), suggesting that the activation domain resides within the C-terminus region spanning amino acids 154 to 255, which is important for activation activity of *CmHRE2-like*.

### Expression profiles of *CmHRE2-like* in the chrysanthemum

**Fig. 3:**
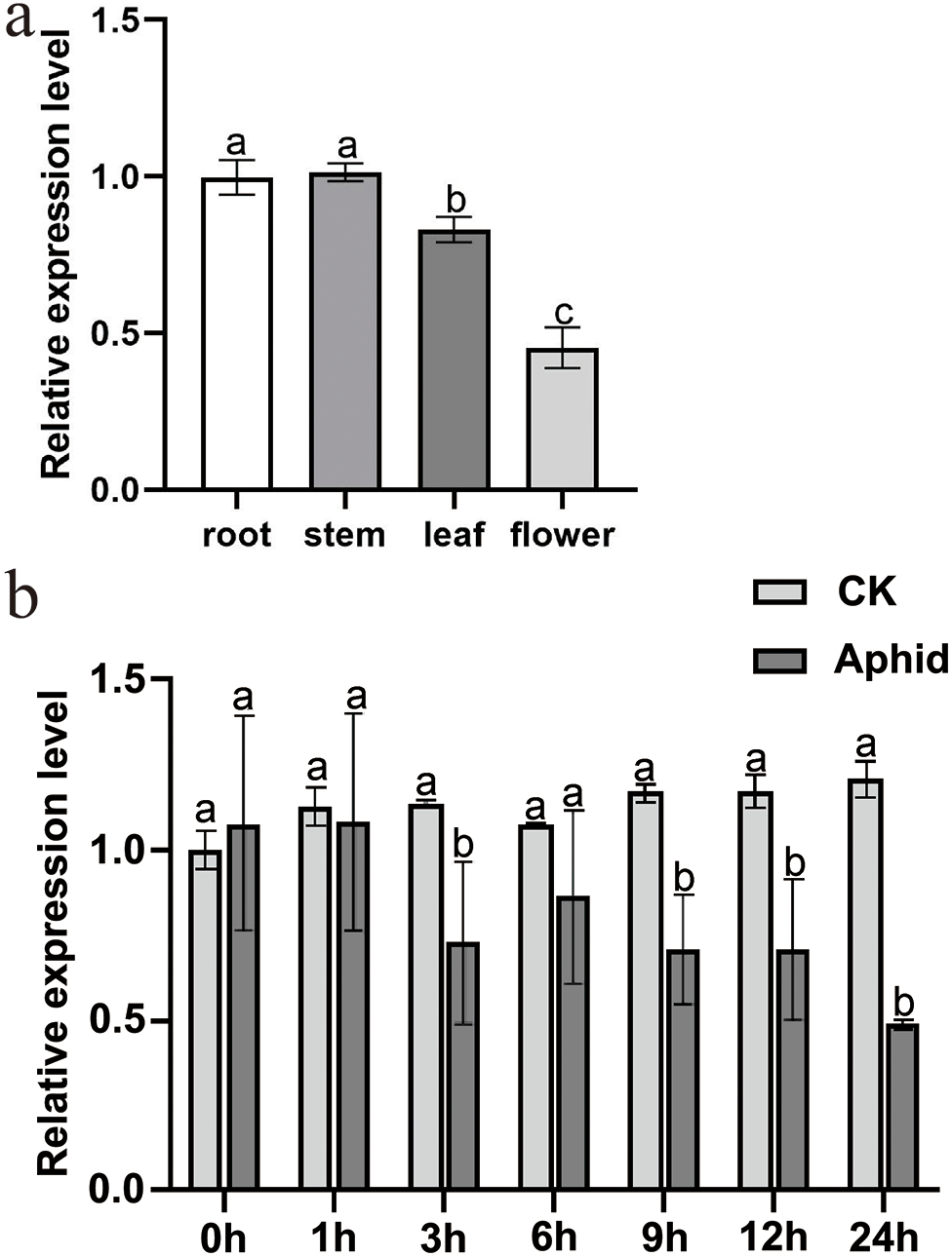
Relative expression pattern of *CmHRE2-like*. **(a)** Relative expression level of *CmHRE2-like* in various organs of chrysanthemum ‘Jinba’; **(b)** Transcriptional changes of *CmHRE2-like* in response to aphid infestation, bars indicate the standard errors, the alphabets represent the level of significant difference (*p* < 0.05)

The expression profiles of *CmHRE2-like* in the different chrysanthemum tissues were determined (Fig. 3a). The expression levels of *CmHRE2-like* were highest in stems and roots, followed by leaves, whereas flowers exhibited the lowest expression levels. Elucidating the function of *CmHRE2-like* in chrysanthemum. We examined the transcriptional profile of *CmHRE2-like* in response to aphids. As the duration of aphid infestation increased, the expression levels of *CmHRE2-like* remained consistently lower than compared to the control. A notable reduction in the expression level of *CmHRE2-like* was observed at 3 h and 9–24 h after aphid infestation, with 0.64-fold of those of the control at 3 h, 0.61–fold at 9 h, 0.60-fold at 12 h, and 0.40-fold at 24 h (Fig. 3b).

### *CmHRE2-like* conferred to the vulnerability of chrysanthemum to aphids

**Fig. 4:**
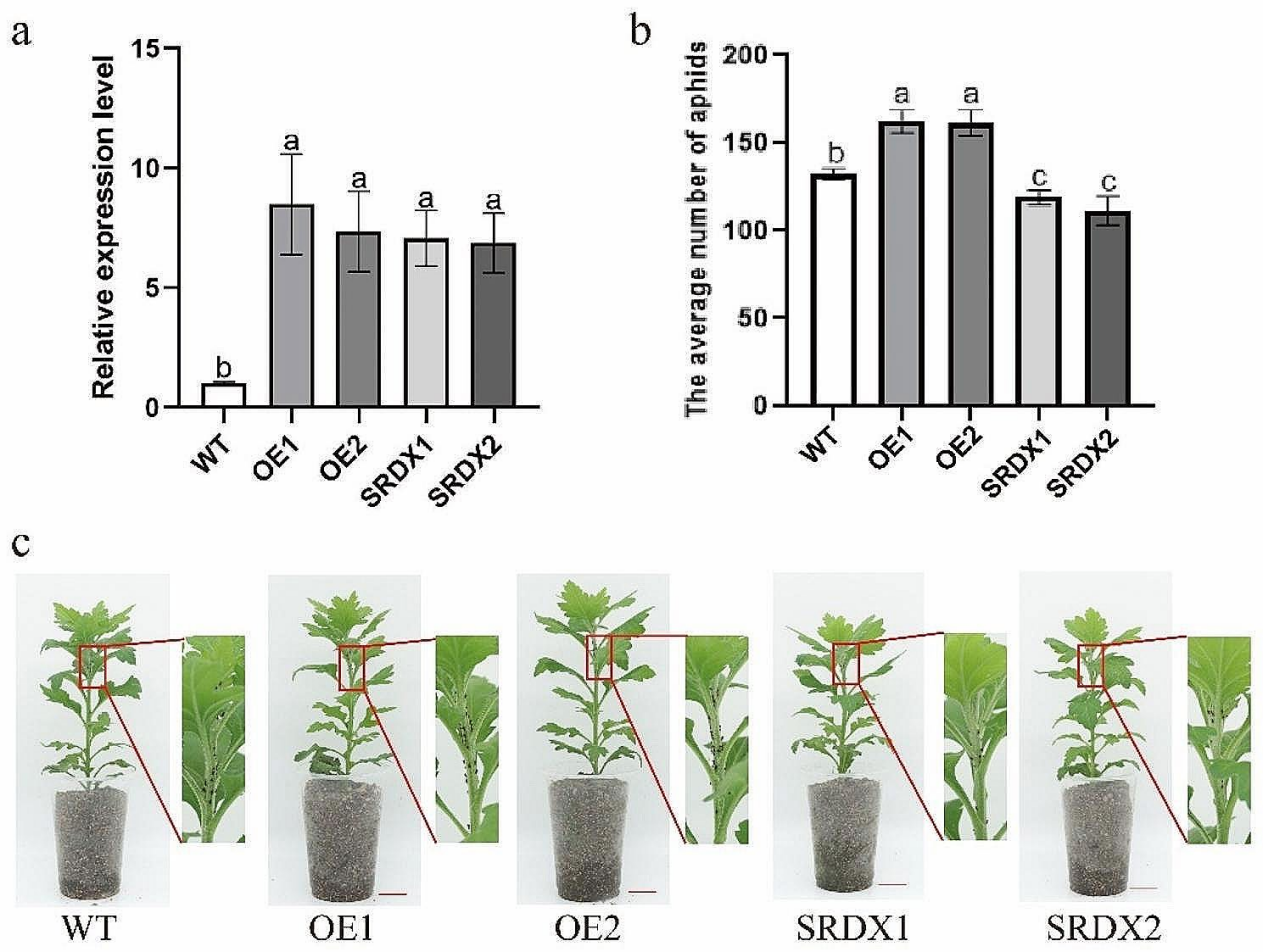
Relative expression levels of *CmHRE2-like* and proliferation of aphids on WT and transgenic lines at 14 days after inoculation, bar = 2 cm. **(a)** Relative expression levels of *CmHRE2-like* in the WT and transgenic plants; **(b)** The average number of aphids present on WT and transgenic lines measured at 14 days after aphid infection. Bars indicate the standard errors; the alphabets represent the level of significant difference (*p* < 0.05); **(c)**. Image of differential proliferation of aphids between WT and transgenic lines

To investigate the function of *CmHRE2-like*, overexpression and dominant suppression of *CmHRE2-like* in transgenic chrysanthemum lines was performed (Fig. 4a). Aphid infestation assays showed that the number of aphids on wild-type (WT) plants was less than that on *CmHRE2-like*-overexpressing plants, while was higher than that on *CmHRE2-like*-SRDX transgenic plants (Fig. 4b). WT plants exhibited an aphid multiplication rate (MR) of 26.48%, whereas it reached 32.46% and 32.32% on *CmHRE2-like*-overexpressing lines OE1 and OE2, respectively, and dropped to 23.8% and 22.28% on *CmHRE2-like*-SRDX gene-silenced lines SRDX1 and SRDX2, respectively. The inhibition ratios (IRs) for *CmHRE2-like*-overexpressing lines OE1 and OE2 were − 22.58% and − 22.05%, whereas they were 10.12% and 15.86% for the SRDX lines SRDX1 and SRDX2, respectively (Fig. 4c; Table 1), indicating that *CmHRE2-like* contributes to the vulnerability of chrysanthemum to aphids.

**Table 1:** MR and IR percent of aphids in WT and *CmHRE2-like* transgenic lines 14 days after the infestation

	WT	OE1	OE2	SRDX1	SRDX2
MR	26.48	32.46	32.32	23.8	22.28
IR%	0	-22.58	-22.05	10.12	15.86

### Transcriptomic analysis of *CmHRE2-like*-regulated gene expression profiles in chrysanthemum

**Fig. 5:**
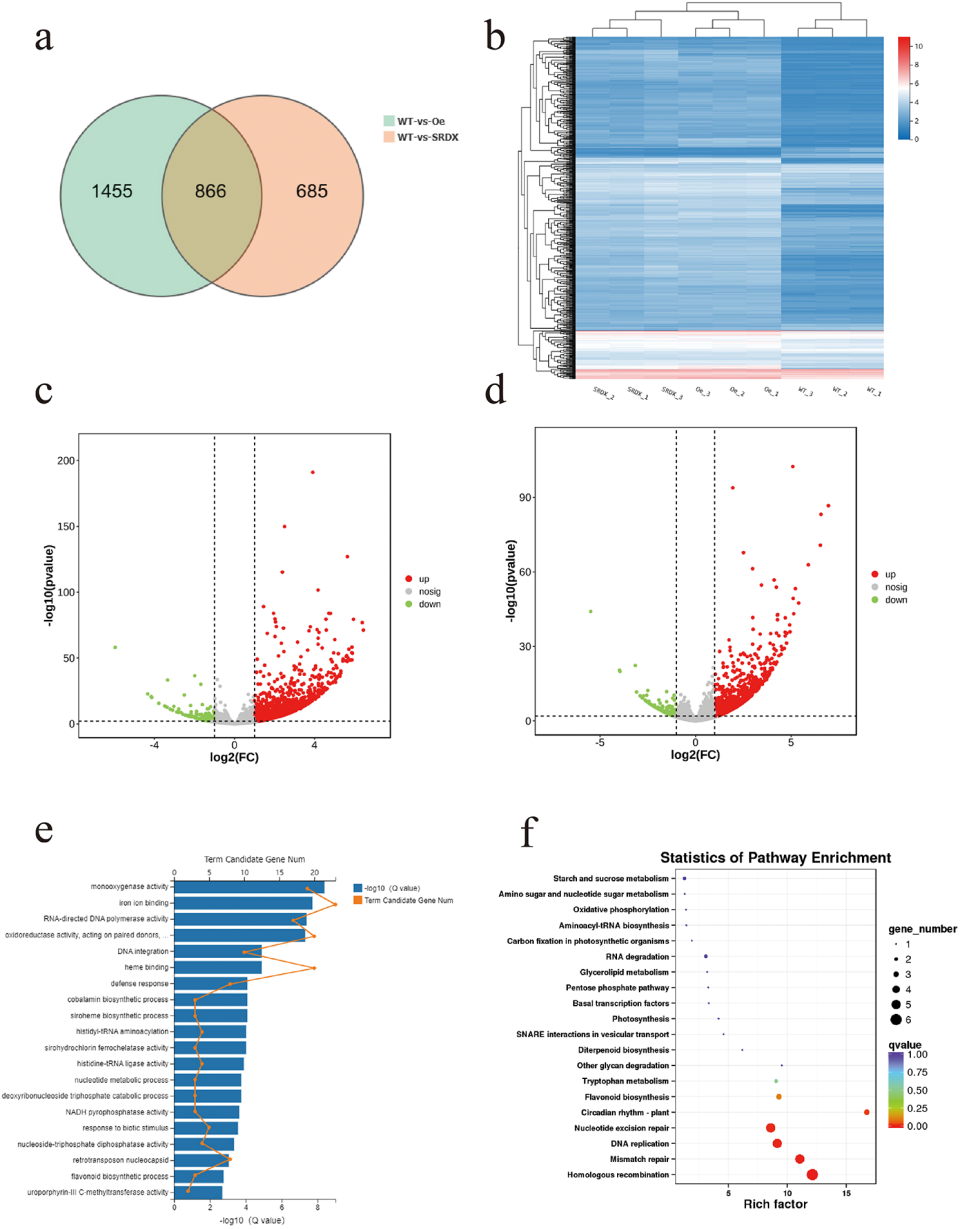
Gene expression and pathway enrichment in transgenic plants and WT plants. **(a)** Venn diagram of DEGs of the comparison between transgenic plants and WT plants. **(b)** Expression profiles derived from RNA-seq are depicted in the cluster; showcasing expression patterns for a subset of genes characterized by high log2 (fold change, FC) values in comparisons between transgenic plants and WT plants, each sampled in triplicate. **(c)** Distribution and expression levels of differentially expressed genes (DEGs) of overexpression lines and WT plants, the log2 (FC) based on the difference between groups was taken as the abscissa, the negative value of the logarithm based on the difference significance test Q-value value of 10-log10 (q) is the ordinate. **(d)** Distribution and expression levels of DEGs of SRDX lines and WT plants. **(e)** Most enriched Gene Ontology (GO) terms of DEGs between *CmHRE2-like* overexpression lines SRDX lines. The length of the X-axis column corresponds to the size of the Q-value value, while the position on the line above the X-axis represents the count of DEGs annotated to the GO term. **(f)** KEGG pathway analysis of DEGs between *CmHRE2-like* overexpression lines and SRDX lines. The graph features the enrichment ratio on the X-axis and the KEGG Pathway on the Y-axis. Bubbles’ sizes convey the number of genes annotated to a KEGG Pathway, while color intensity reflects the enrichment Q-value, with deeper colors denoting lower Q-value value. The copyright for KEGG images has been obtained through written permission from Kanehisa Laboratories

In order to clarify the potential mechanism through which *CmHRE2-like* exerts a negative regulatory effect on defense responses in chrysanthemum, we analyzed *CmHRE2-like* transgenic plants. When comparing *CmHRE2-like* overexpression lines and WT plants, our analysis revealed 2,321 differentially expressed genes (DEGs), while 1,551 DEGs were found when comparing SRDX lines to WT plants (Fig. 5a). Gene expression levels of DEGS in transgenic and WT plants were assessed using log2 (fold change, FC) values, as represented in a heatmap (Fig. 5b). The cluster analysis results showed that expression data for all groups showed good repeatability. Volcano plots exhibited the distribution of fold changes, and the Q-values are shown in Fig. 5c and d. A total of 1,899 DEGs were upregulated and 422 were downregulated between the overexpression lines and WT plants. In contrast, 1,264 DEGs were upregulated and 287 were downregulated in the SRDX lines and WT plants.

To classify the functions of the DEGs, we performed Gene Ontology (GO) enrichment analysis, the data revealed that a significant proportion of genes were linked to pathways associated with various stress responses, including defense responses, responses to biotic stimuli, and flavonoid biosynthetic processes (Fig. 5e). We conducted a pathway enrichment analysis to explore the biological pathways linked to the DEGs (Fig. 5f). Our KEGG pathway enrichment analysis indicated that DEGs that were differentially expressed in the transgenic plants were associated with plant-pathogen interactions, including diterpenoid biosynthesis, other glycan degradation, and flavonoid biosynthesis.

### *CmHRE2-like* inhibits the production of total flavonoids

**Fig. 6:**
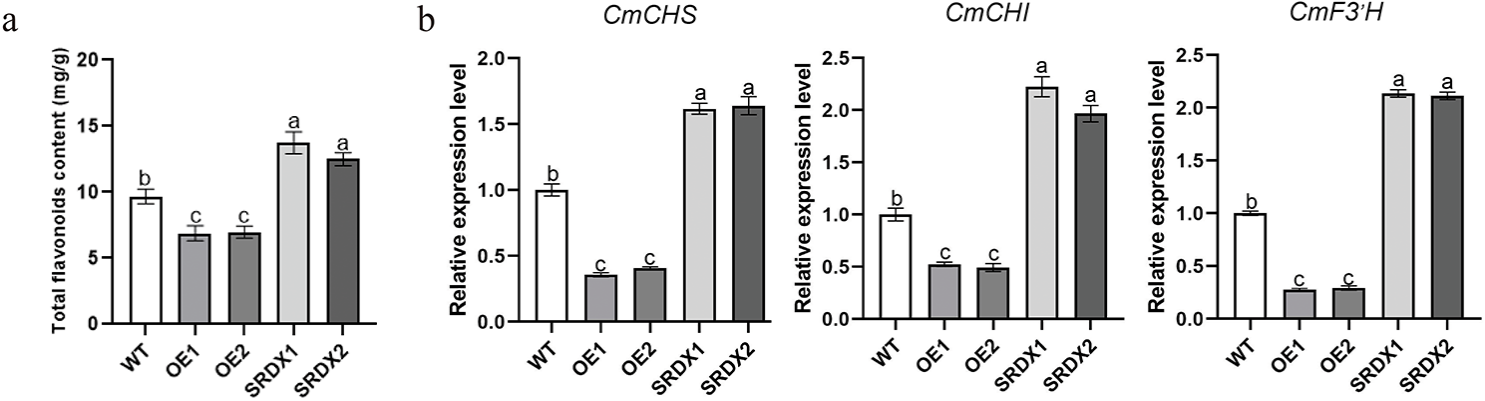
Analysis of the flavonoid biosynthesis between transgenic plants and WT plants. **(a)** Determination of total flavonoid content in WT plants and transgenic plants; **(b)** Differentially expressed genes (DEGs) involved in the flavonoid biosynthesis between WT plants and transgenic plants, bars indicate the standard errors, the alphabets represent the level of significant difference (*p* < 0.05)

Based on the transcriptomic data, we postulate that the regulatory role of *CmHRE2-like* in chrysanthemum aphid resistance is partially mediated by the regulation of flavonoid biosynthesis. To validate this hypothesis, we detected the total flavonoid content in the *CmHRE2-like* transgenic lines and WT plants. The content in the overexpression lines exhibited a significant reduction compared to the content in the WT, whereas the SRDX lines exhibited a marked increase in total flavonoid content (Fig. 6a). The total flavonoid content of *CmHRE2-like*-overexpressing lines OE1 and OE2 decreased by 0.71-fold and 0.72-fold, respectively, compared to the WT plants, whereas *CmHRE2-like*-SRDX lines SRDX1 and SRDX2 increased by 1.42-fold and 1.29-fold, respectively, compared to the WT plants. These findings suggest that *CmHRE2-like* modulates aphid resistance in chrysanthemum by regulating flavonoid content. Additionally, quantitative reverse transcription-polymerase chain reaction (qRT-PCR) served as the method of choice for determining the transcription levels of genes participating in flavonoid biosynthesis. Our analysis demonstrated that the transcript levels of three genes (*CmCHS*, *CmCHI*, and *CmF3’H*) associated with flavonoid biosynthesis were elevated in SRDX-*CmHRE2-like* plants but reduced in the overexpression plants in comparison to WT plants (Fig. 6b). The findings of our qRT-PCR analyses aligned with the outcomes from the RNA-seq data.

## Discussion

### *CmHRE2-like* negatively regulated the resistance of chrysanthemum to aphids

AP2/ERF family TF have been identified as key regulators of various stress responses [21, 22], and HRE2, a member of the TF family, holds a significant position in mediating plant tolerance to abiotic stress. Several studies have indicated that HRE2 affects the development of adventitious roots in plants to regulate waterlogging tolerance [23–25]. Additionally, in *Arabidopsis*, *GsERF071* regulates H^+^-ATPase activity and modifies auxin accumulation in roots, which functions as a positive modulator enhancing alkaline stress tolerance [26]. Bioinformatic analysis indicated that *OsERF071* orchestrates a specific modulation of gene expression to produce transgenic rice with a drought-resistant phenotype, suggesting that *OsERF071* channels a greater portion of available resources towards indispensable mechanisms like translation, oxidative responses, and DNA replication [27]. Nevertheless, there have been few investigations into the role of HRE2s in the modulation of resistance to biotic stress. In this investigation, we report for the first time that *CmHRE2-like* exerts a negative influence on the resistance of chrysanthemum to aphids, suggesting that HRE2 may exhibit functional variations across diverse species.

### *CmHRE2-like*-altered aphid resistance is potentially related to flavonoid biosynthesis

Flavonoids play a significant role as major polyphenolic compounds in plant secondary metabolism [28]. Previous studies have highlighted the effects of flavonoids on aphid survival. Flavonoid accumulation has been observed in the epidermal cells of pea seedlings following aphid infection [29]. Additionally, a positive correlation was observed between the resistance/susceptibility traits of aphids and flavonoid glycoside content of cowpea lines, with resistant lines displaying elevated flavonoid content than susceptible lines [4]. Nevertheless, there is limited knowledge regarding the molecular mechanisms underlying flavonoid biosynthesis in chrysanthemum when exposed to aphid infection.

The AP2/ERF superfamily of TFs regulates flavonoid biosynthesis by modulating genes responsible for metabolite biosynthesis [30]. For instance, the *MdAP2-34* was found to promote flavonoid accumulation by directly binding to and activating the MdF3′H promoter in apples, thus promoting the flavonoid biosynthetic pathway [31]. Similarly, melatonin promotes the accumulation of flavonoids by suppressing the inhibition of *BrERF2/BrERF109* on the transcripts of *BrFLS1* and *BrFLS3.2*, ultimately postponing leaf senescence in postharvest flowering Chinese cabbage [32]. Additionally, through its interaction with *MdMYB1*, *MdERF38* facilitates the binding of *MdMYB1* to its target genes, thus augmenting anthocyanin biosynthesis in response to drought stress [33]. In the present study, a substantial decrease was evident in total flavonoid content in the overexpression lines compared to the WT, whereas the SRDX lines exhibited a marked increase in total flavonoid content. Furthermore, in our analysis, we observed an increase in the transcript levels of three flavonoid biosynthesis genes (*CmCHS*, *CmCHI*, *CmF3’H*) in SRDX-*CmHRE2-like* plants whereas they displayed a decrease in overexpression plants in comparison to the WT plants. Thus, we propose that *CmHRE2-like* may mediate vulnerability to aphids through the regulation of plant flavonoid biosynthesis; however, the detailed mechanisms by which HRE2-like regulates chrysanthemum biosynthesis remain to be elucidated. In addition, genes related to ‘defense response’ and ‘response to biotic stimulus’ were differentially expressed in *CmHRE2-like* transgenic plants, suggesting that CmHRE2-like may regulate pathways to cope with aphid feeding, which will be our ongoing research.

## Conclusion

In this investigation, we isolated an ERF TF *CmHRE2-like*, and its molecular characterization was investigated in chrysanthemum. The results showed that overexpressing of *CmHRE2-like* increased the vulnerability of chrysanthemum to aphids, whereas its SRDX transgenic plants increased the resistance to aphids. During the defense response to aphids, *CmHRE2-like* was negatively associated with the flavonoid biosynthesis in chrysanthemum. The transcript levels of three flavonoid biosynthesis genes (*CmCHS, CmCHI, CmF3’H*) were increased in SRDX-Cm*HRE2-like* plants but decreased in the overexpression plants when contrasted with the WT plants. The data generated in this study indicate that *CmHRE2-like* is a TF that with a role in determining chrysanthemum’s vulnerability to aphids by governing the biosynthesis of flavonoids in the plant.

## Materials and methods

### Plant materials and treatment

Cuttings of the chrysanthemum varieties ‘Jinba’ was obtained from the Chrysanthemum Germplasm Resource Preserving Center at Nanjing Agricultural University, Nanjing, China [25]. The plants were transferred into pots containing a blend of nutrient-enriched soil and vermiculite, with a volumetric ratio of 1:3 (soil to vermiculite).Under controlled greenhouse conditions, the plants were raised with a 70% relative humidity, a photoperiod of 16 h with 8 h of darkness [25], and day-night temperatures maintained at 23 °C and 18 °C. The light was calibrated to an intensity of 100 µmol m^− 2^ s^− 1^. Both stems and leaves served as the source materials for RNA extraction [15].

For the phytohormone treatment, Seedlings were subjected to suspensions containing 100 mg/L of Ethephon (Solarbio, Beijing, China) in deionized water, applied using a handheld sprayer until the solution started dripping off the leaves. Leaf and stem samples were collected at 0, 1, 3, 6, 9, 12, and 24 h after treatment initiation [34]. Three individual plant replicates per sample were used to collect portions above the second leaf, and the experiment was conducted thrice [19].

For the analysis of gene expression profiles in response to aphid feeding, we introduced 20 aphids onto the apical buds and young stems of plants at the 6–8 leaf-stage chrysanthemum. Aphids (*Macrosiphoniella sanbourni*) were collected from chrysanthemum ‘Jinba’ plants, second instar nymphs were chosen to infect plants [35]. The isolation of RNA entailed the collection of samples from the three inoculated plants at time intervals of 0, 1, 3, 6, 9, 12, and 24 h [19]. Each sample consisted of three individual plant replicates and the experiment underwent three repetitions.

Aphid treatments involved infesting plants at the 6–8 leaf stage with five nymphs, and the total aphid count was recorded 14 days subsequent to infestation [19, 35]. Plant resistance was quantified using the Metrics of Resistance (MR) and Infestation Rate (IR). MR was calculated as Ni/5, with Ni representing the total aphid population on each chrysanthemum after 14d aphid infection, and IR was defined as 100(NW-NT)/NW, where NT and NW indicated the mean aphid numbers recorded 14 d after aphid treatment in transgenic and WT plants, respectively [15, 19]. Ten plants from each line constituted a single treatment, and the study encompassed three biological replicates.

### Gene isolation and phylogenetic analysis

RNAiso reagent (TaKaRa, Tokyo, Japan) was used to extract total RNA from leaves, followed by reverse transcription using Reverse Transcriptase M-MLV (TaKaRa). Primers HRE2-like F/R were employed to clone the open reading frame(ORF) of using PCR. After purification, a pMD19-T vector (TaKaRa Bio) was employed to clone the PCR product, enabling subsequent sequencing. DNAMAN 6 software was used to conduct multiple sequence alignments of *CmHRE2-like* and its homologs. A phylogenetic tree crafted via using MEGA 5 software (available on: http://www.megasoftware.net) employing the neighbor-joining method and 1,000 bootstrap replicates [36]. Polypeptide sequences of ERF071 homologs were obtained from the National Center for Biotechnology Information website (https://www.ncbi.nlm.nih.gov) [37].

### qRT-PCR

Relative expression levels were detected using qRT-PCR. Isolation and reverse transcription of total RNA were followed by the utilization of SYBR® Premix Ex Taq™ II (Tli RNaseH Plus; Takara) in 20 µL reactions according to the manufacturer’s instructions. Then the qPCR was performed using the LightCycler96 Real-time PCR System. The qPCR amplification was performed in thermocycler conditions starting with 30 s at 94 °C, 5 s at 94 °C, 40 cycles of 30 s at 60 °C, and 10 s at 95 °C, followed by 1 min at 60 °C. Chrysanthemum gene elongation factor 1α (*CmEF1α*, KF305681) was employed as a reference in the data analysis using the ^2ΔΔC^_T_ method [17]. Each qRT-PCR was conducted in triplicate. Gene-specific primers were designed by Primer Premier 5, and they are listed in Supplementary Table S1.

### Transactivation assays

Transactivation assays were performed as described previously [38]. Cloning of both the coding region and truncated sequences of CmHRE2-like was accomplished into the pDEST-GBKT7 vector [15], followed by the transformation of resulting pDEST-GBKT7-*CmHRE2-like* fusion plasmids into the yeast strain Y2H Gold (Clontech, Mountain View, CA, USA). Positive and negative controls were introduced as plasmids of pCL1 and pDEST-GBKT7, respectively. Transformant cultures were distributed onto SD/-Trp medium, whereas the pCL1- carrying strain was cultivated on SD/-Leu medium. Subsequently, the colonies were then relocated to SD/-His/-Ade media and subjected at 30 °C for 3 d to assess the transactivation assays [15].

### Subcellular localization of CmHRE2-like

The *CmHRE2-like* ORF was inserted into the pMDC43 vector, forming a construct where the *CmHRE2-like* ORF was linked to GFP at the N-terminus [39]. Following this, the generated plasmids were delivered into the *A. tumefaciens* strain GV3101 [40]. To examine the expression of *CmHRE2-like*, co-transformation of *N. benthamiana* leaves involved 35 S::GFP *CmHRE2-like* and 35 S::D53-RFP constructs. The nuclear marker within the 35 S::D53-RFP plasmid consisted of a fusion between the red fluorescent protein (RFP) and D53 protein. Observations of RFP and GFP expression were conducted utilizing a Zeiss LSM800 Ultra high resolution confocal microscope (Germany).

### Generation of *CmHRE2-like* transgenic chrysanthemum

In this experiment, cloning of the*CmHRE2-like* sequence commenced with its insertion into a pENTR1A gateway vector, which was subsequently transferred to a pMDC43 overexpression vector [19]. The resulting construct was driven by the 2 × 35 S promoter. The creation of a dominant repressor involved the fusion of *CmHRE2-like* with the EAR repression domain (SRDX), which converts transcriptional activators into strong repressors [41–44]. After the generation of the *CmHRE2-like*-SRDX construct, it was incorporated into the *A. tumefaciens* EHA105 strain and employed for chrysanthemum transformation via Agrobacterium-mediated means [45]. Verification of transgenic plants involved PCR analysis utilizing both vector- and gene-specific primers, alongside the quantification of *CmHRE2-like* expression through qRT-PCR using *CmHRE2-like*-RT-F/R primers [15]. Table S1 contains a record of the primers used in this study.

### Transcriptome analysis

The experiment involved the use of *CmHRE2-like*-overexpressing plants (*CmHRE2-like*-OE1), *CmHRE2-like*-SRDX transgenic plants (*CmHRE2-like*-SRDX1), and WT plants at the 6–8-leaf stage [17]. Samples for further analysis were obtained from the portions situated above the second leaf from the apex of each plant. Each experiment comprised three biological replicates, each containing nine plants. Total RNA was extracted from the collected leaf samples and 1.5 µg of RNA was used for RNA-seq library preparation and generation (BGI, China) [15]. RNA-Seq data were analyzed using the DESeq method, with a screening threshold for differential gene expression was set at padj < 0.05 [46]. Conducting transcriptome data assembly and analysis using BGI Dr.Tom Cloud Platform (https://report.bgi.com/) [47, 48].

### Statistical analysis

The statement above describes the statistical methods used in this study. Statistical analysis was performed using SPSS 20.0 software, and the means ± standard errors were used to express the results for both WT and transgenic plants [49]. Statistical significance was assessed through One-way analysis of variance (ANOVA), and the results were further analyzed using the least significant difference (LSD) multiple range test [15].

### Determination of total flavonoids content

Total flavonoid content was quantified using a commercial flavonoid assay kit (Solarbio, Beijing, China) following the guidelines provided by the manufacturer [19]. Each line was represented by three independent plant replicates and the experiment was performed in triplicate.

### Electronic supplementary material

Below is the link to the electronic supplementary material.

Supplementary Material 1

## Data Availability

The raw transcriptome data used during this study has been deposited in NCBI SRA with the accession number PRJNA953678.
