# Supplementary material for: Chrysanthemum (Chrysanthemum morifolium) CmHRE2-like negatively regulates the resistance of chrysanthemum to the aphid (Macrosiphoniella sanborni)
Source: BMC Plant Biol. 2024 Jan 29;24:76. doi: 10.1186/s12870-024-04758-6 (PMC10823704; doi:10.1186/s12870-024-04758-6)
Supplement: Supplementary file 1 — Supplementary Material 1 [file 12870_2024_4758_MOESM1_ESM.docx]

**Supplementary materials**

**Table S1** Primer sequences used in the paper

| primer | sequence |
| --- | --- |
| CmHRE2-LIKE-ORF-F | ATGTGCGGTGGTGCAATTCTT |
| CmHRE2-LIKE-ORF-R | TTACACAACATCATCAAGATC |
| CmHRE2-LIKE--RT-F | TGTCTGAGGAGCTAATGGCGTATGA |
| CmHRE2-LIKE--RT-R | ATCACCACCACCACCACCATCA |
| CHI-RT-F | GAGCGTCCGTTGTTCGAGATGATAT |
| CHI-RT-R | GAAATGTTAGCCCGACTTCCCTCAA |
| CHS-RT-F | GAGCGTCCGTTGTTCGAGATGATAT |
| CHS-RT-R | GAAATGTTAGCCCGACTTCCCTCAA |
| F3’H-RT-F | ACCGGCTTGTAACAGAATTGGACTT |
| F3’H-RT-R | TCTTGGTAAGGAGAGTGGCGTTGA |
| 35S-F | GACGCACAATCCCACTATCC |
| SRDX-R | AGCGAAACCCAAACGGAGTTCTAG |
| EF1A-F | TTTTGGTATCTGGTCCTGGAG |
| EF1A-R | CCATTCAAGCGACAGACTCA |

**Table S1** Primer sequences used in the paper
